# The Effect of Prickly Ash (*Zanthoxylum bungeanum* Maxim) on the Taste Perception of Stewed Sheep Tail Fat by LC-QTOF-MS/MS and a Chemometrics Analysis

**DOI:** 10.3390/foods10112709

**Published:** 2021-11-05

**Authors:** Yan Huang, Dandan Pu, Zhilin Hao, Xiao Yang, Yuyu Zhang

**Affiliations:** Beijing Key Laboratory of Flavor Chemistry, Beijing Technology and Business University, Beijing 100048, China; huangyan_916@163.com (Y.H.); 18518351472@163.com (D.P.); hzl15716324037@163.com (Z.H.); yangxiao@btbu.edu.cn (X.Y.)

**Keywords:** sheep tail fat, taste compounds, LC-QTOF-MS, PLS-DA, sensory analysis, additional experiment

## Abstract

This work aims to explore the contribution of prickly ash (*Zanthoxylum bungeanum* Maxim) on the taste perception of stewed sheep tail fat. Liquid chromatography-tandem quadrupole time of flight mass spectrometry (LC-QTOF-MS) was applied to analyze the taste-related compounds. A total of 99 compounds in different sheep tail fat samples were identified. The semi-quantitative results showed that there were differences between the samples. The partial least squares discriminant analysis (PLS-DA) model without overfitting was used to investigate the effect of prickly ash. Eleven marker compounds were predicted with a variable importance for projection > 1, fold change > 2 and *p* < 0.05. An additional experiment showed that guanosine 5′-monophosphate, malic acid, inosine and adenosine 5′-monophosphate could improve the umami and saltiness taste of stewed sheep tail fat.

## 1. Introduction

Sheep tail fat is an important part of the sheep with a unique flavor after heat treatment. The weight of the sheep tail fat from individual sheep varies from 3 to 8 kg. In northwest China, stewed sheep tail fat is an essential hotpot additive for many consumers to enhance its taste. However, sheep tail fat use is limited in the food industry due to its widely unaccepted flavor [1,2]. The large backlog of sheep tail fat boosts its application in the food industry. In this case, the crucial flavor problem demands a solution.

Improving the taste of sheep tail fat is an effective way to promote its industrial development and solve the extrusion problem of sheep tail fat. Spices have a long planting history in China and they have been widely used in meat product processing because of their seasoning, flavoring, appetizing and other functions [3,4]. Gourmets improve the overall taste of meat products by adding spices based on different cooking methods [5]. In the research of roasted mutton, spices were found to be an effective additive to change the flavor compounds: the addition of prickly ash (*Zanthoxylum bungeanum* Maxim) and ginger increased the content of terpenes and cumin and amomum tsao-ko increased the content of aldehydes in the roast meat [6]. Although spices enriched the volatile compounds in the roast mutton, they did not weaken the characteristic flavor of the mutton and helped to improve the flavor of the food. Prickly ash has a good application performance in meat products. Adding prickly ash to salted silver carp can reduce the content of volatile fishy compounds such as benzene, methylbenzene, 1-octene-3-ol, hexanal and methyl ketone, thus improving the flavor of the salted fish [7]. The addition of prickly ash can also improve the overall taste by promoting the release of taste compounds. Bi et al. studied the effect of prickly ash on the flavor influence of ham during storage. The results showed that the addition of pepper resulted in changes of the taste compounds such as amino acids, sugars, organic acids and nucleotides, which provided a new taste balance to the ham with a light overall taste that was beneficial to a group of people [8].

In order to fully understand the flavor characteristics of the research sample, a greater number of compounds need to be identified for the subsequent analysis. Mass spectrometry has been widely used in food flavor analyses due to its high-resolution ratio and sensitivity [9]. A large amount of data were produced during the data analysis, which are valuable for providing helpful information. An increasing number of researchers have applied the chemometric method to correlate the relationship of chemical parameters to the sensory qualities to discover compounds contributing to sensory attributes [10,11,12]. Non-targeted metabonomic technology combined with liquid chromatography-mass spectrometry (LC-MS) was used to compare the metabolic characteristics of meat samples of Lubei white goats, Boer goats and Jining Grey goats. The results of a partial least squares discriminant analysis (PLS-DA) and an orthogonal partial least squares discriminant analysis (OPLS-DA) showed that there were significant differences in the fatty acids, aldehydes, ketones, lactones, alkaloids, flavonoids, phenols and drug residues among the three kinds of meat, which might have been the source of their flavor differences [13]. A gas chromatography-mass spectrometer (GC-MS) combined multivariate data analysis also demonstrated a good performance. A total of 32 aroma-active compounds were identified by GC-MS in a mutton sample. Based on the fingerprint data, a principal component analysis (PCA) and a PLS-DA, 14 components were predicted to be the main marker compounds [14].

In this work, prickly ash-processed stewed sheep tail fat and stewed sheep tail fat were used as the main research samples to explore the effect of prickly ash on the taste contribution to the sheep tail fat. The compounds in all samples were identified by liquid chromatography-tandem quadrupole time of flight mass spectrometry (LC-QTOF-MS/MS) and the compounds were semi-quantified by internal standard compounds. A reliable PLS-DA model was established based on a permutation test and cross-validation and marker compounds were selected through variable importance in projection (VIP) > 1, fold change (FC) > 2 and *p* < 0.05. The taste characteristics of the marker compounds were verified by an additional experiment and an electronic tongue.

## 2. Materials and Methods

### 2.1. Materials and Chemicals

Sheep tail fat (Sunit sheep) and prickly ash (powder) were purchased from Xilingol League, Inner Mongolia and Guangxi province, respectively. Methanol with an MS grade was purchased from Merck Chemical Technology Co. Ltd. (Shanghai, China). Ammonium formate and formic acid with an MS grade were purchased from Bailingwei Technology Co. Ltd. (Beijing, China). Ultrapure water was purchased from Watson’s food and beverage Co. Ltd. (Guangzhou, China); flavone, L-theanine, 1,2-dichlorobenzene, methyl palmitate, silibinin and gamma-nonanolactone with an analytical grade were all purchased from Aladdin biochemical technology Co. Ltd. (Shanghai, China) and guanosine 5′-monophosphate (5′-GMP), malic acid, inosine, adenosine 5′-monophosphate (5′-AMP), guanosine, fumaric acid and 18β-glycyrrhetinic acid with an analytical grade were purchased from Sigma-Aldrich (St. Louis, MO, USA).

### 2.2. Sample Preparation

According to the pre-experiment results in our group, the optimal technology of stewing sheep tail fat was a solid–liquid ratio of 1:2 and a stewing time of 3 h. Therefore, to explore the effect of prickly ash on stewed sheep tail fat, stewed sheep tail fat with 0.2% prickly ash (SP) was studied; stewed sheep tail fat (S) and stewed prickly ash (P) served as the controls. Each sample was repeated three times.

After stewing, the fat blocks and the liquids were separated with gauze then the liquids were transferred to the separating funnel. The water phase and the oil phase were then separated and finally we obtained three parts: soup (OP); the fat block (OB); and the upper oil layer (OU). The fat block was placed in a beaker, mixed with 50% edible alcohol in the ratio of 1:1 and ultrasonicated for 30 min at 400 W. After ultrasonic treatment, it was filtered with gauze and the filtrated liquid was transferred into a separating funnel. After the separation, the water phase was collected to obtain the sample. The upper oil layer was placed in a beaker, mixed with 50% edible alcohol at a ratio of 1:1 and ultrasonicated for 15 min at 400 W. After ultrasonic treatment, this was filtered with gauze and the filtrated liquid was transferred into a separating funnel. After the separation, the water phase was collected to obtain the sample. Finally, we obtained three group of samples: soups (with prickly ash, SP-OP; without prickly ash, S-OP); fat blocks (with prickly ash, SP-OB; without prickly ash, S-OB); and an upper oil layer (with prickly ash, SP-OU; without prickly ash, S-OU).

### 2.3. Sensory Evaluation

The panelists of sensory evaluation were recruited from the laboratory and received systematic taste training. Panelists who could distinguish five basic tastes were selected. A total of 12 panelists (4 males and 8 females, aged 20–30) were selected to evaluate the samples. According to previous results [15,16,17], the food-grade taste solutions including sodium glutamate (0.08 g/100 mL), sodium chloride (0.30 g/100 mL), quinine (0.00075 g/100 mL), sucrose (2.00 g/100 mL) and citric acid (0.05 g/100 mL) represent umami, saltiness, bitterness, sweetness and sourness, respectively, with a score of 5 (total scale of 1–9 where 1 is weak; 5 is medium and 9 is strong).

In order to explore the contribution of prickly ash to the taste of stewed sheep tail fat, S, P and SP samples (55 °C, optimized in our laboratory) were simultaneous presented to the panelists to evaluate the taste profiles in the sensory evaluation laboratory (22 °C, 60% humidity). The three samples were coded with three digital numbers and were submitted to the panelists for sensory evaluation in a random order. The panelists scored the five basic taste intensities by referencing the standard reference compounds. The overall taste was evaluated according to the preference of the panelists. Each sample was repeated three times.

### 2.4. Electronic Tongue

An electronic tongue (SA-402B, Insent) was used to analyze the taste characteristics including umami, sweetness, saltiness, sourness and bitterness. The sensors were soaked in a reference solution (30 mM KCl solution with 0.3 mm tartaric acid) for more than 24 h including AAE (umami), GL1 (sweetness), CT0 (saltiness), CA0 (sourness) and C00 (bitterness) coupled to a reference electrode (Ag/AgCl). Before the determination, the instrument was calibrated and initialized to ensure the stability and reliability of the data. The injection procedure was set as the sample taste collection time (30 s) and the cleaning time (300 s). Each sample was repeated three times.

### 2.5. Detection Method

The compound analysis was performed on a UPLC-ESI-QTOF system (AB Sciex, Framingham, MA, USA). Chromatography separation was achieved on a Kinetex^®^ C18 column (100 × 4.6 mm, 2.6 μm) with a flow rate of 0.6 mL/min, an injection volume of 10 μL and a column temperature of 40 °C in both ESI^+^ and ESI^−^ modes. For ESI^+^, the mobile phase consisted of A (0.1% formic acid in water) and B (0.1% formic acid in acetonitrile); for ESI^−^, the mobile phase consisted of A (20 mM ammonium formate in water) and B (0.1% formic acid in acetonitrile). A gradient elution was carried out using 0–33.0 min, 5–18% B; 33.1–46.0 min, 35% B; 46.1–49.0 min, 80% B; 49.1–53.0 min, 80% B; 53.1–54.0 min, 95% B; and 54.1–60.0 min, 5% B.

The mass spectrometric data were collected on a SCIEX X500R QTOF mass spectrometer (AB Sciex, Framingham, MA, USA). The TOF-MS and TOF-MS/MS were scanned with a mass range of *m/z* 50–1000. A dynamic background subtraction (DBS) trigger information-dependent acquisition (IDA) was used to obtain the MS/MS data. The maximum candidate ion number of 15 was selected and the intensity threshold was set as 100 cps. The electrospray ion source temperature and the spray voltage were set to 550 °C, 5500 V and 550 °C, −4500 V for the ESI^+^ and ESI^−^ modes, respectively. The declustering potential (DP), collision energy (CE), collision energy spread (CES), gas 1, gas 2 and curtain gas were 80 V, 35 V, ±15 V, 55 psi, 60 psi and 35 psi, respectively. A continuous recalibration was carried out after every ten samples with CDS tuning fluid and one quality control (QC) sample (an equal amount of each sample was evenly mixed) was inserted for every five samples. The accurate mass and composition of the precursor ions and fragment ions were obtained. The compounds was analyzed by the MassBank database (https://massbank.eu/MassBank/ (accessed on 20 August 2021)) and subsequently ensured by an ion fragment from the standard compounds.

When considering the different properties of the compounds, it should be noted that a few compounds can only be detected in the ESI^+^ mode, others can only be detected in the ESI^−^ mode and a few compounds can be detected in both ESI^+^ and ESI^−^ modes. To facilitate the subsequent data analysis, the identified compounds were semi-quantified and six compounds were selected as the internal standards: flavone; L-theanine; 1,2-dichlorobenzene; methyl palmitate; silibinin; and gamma-nonanolactone. The semi-quantitative compounds by different internal standards are shown in Supplementary Materials Table S1.

### 2.6. Additional Experiment

According to the analysis results of the PLS-DA, the marker compounds of the three group samples (soups, fat blocks and upper oil layers) were selected. In addition, 7 compounds were selected for a subsequent analysis that might contribute to taste according to previous works [15,18]. The marker compounds were analyzed by the electronic tongue and added to the S-OP sample to verify its taste improvement effect. The difference between the samples determined the additional concentration of the compounds; that is, the content of the SP samples minus the content of the S samples provided the concentration of the compound to be added. However, several compounds present different taste characteristics at different concentrations such as no taste change at a low concentration, a promotion at a medium concentration and an inhibition at a high concentration. Therefore, in this experiment, the compounds with different concentration differences (5.0, 2.0, 1.0, 0.5 and 0.2 times) were selected for the taste analysis and an additional experiment. The concentrations of the different multiples are shown in Table S2.

### 2.7. Multivariate Data Analysis

Before performing the multivariate statistical analysis, a semi-quantitative data file to MetaboAnalyst 4.0 (https://www.metaboanalyst.ca/ (accessed on 7 September 2021)) was uploaded. Subsequently, the data were pre-processed including the estimate missing value (KNN), the normalized by mean-centered and the pareto-scaled data. The PLS-DA model was used to select the metabolites and separate the groups (soup groups, fat block groups and upper oil layer groups) with the parameter of VIP > 1, FC > 2 and *p* < 0.05. A cross-validation (*R*^2^ and *Q*^2^) was used to verify the stability of the model and a permutation test (1000 cycles) was used to prove that the model was not overfitting [19].

### 2.8. Statistical Analysis

The mass spectrometry data acquisition and processing were based on SCIEX OS software 1.5 (AB Sciex, Framingham, MA, USA). A statistical analysis of the electronic tongue data was performed using Microsoft Excel. The PCA and PLS-DA models were carried out by MetaboAnalyst (https://www.metaboanalyst.ca/ (accessed on 7 September 2021)).

## 3. Results and Discussion

### 3.1. Sensory Evaluation Results

**Figure 1:**
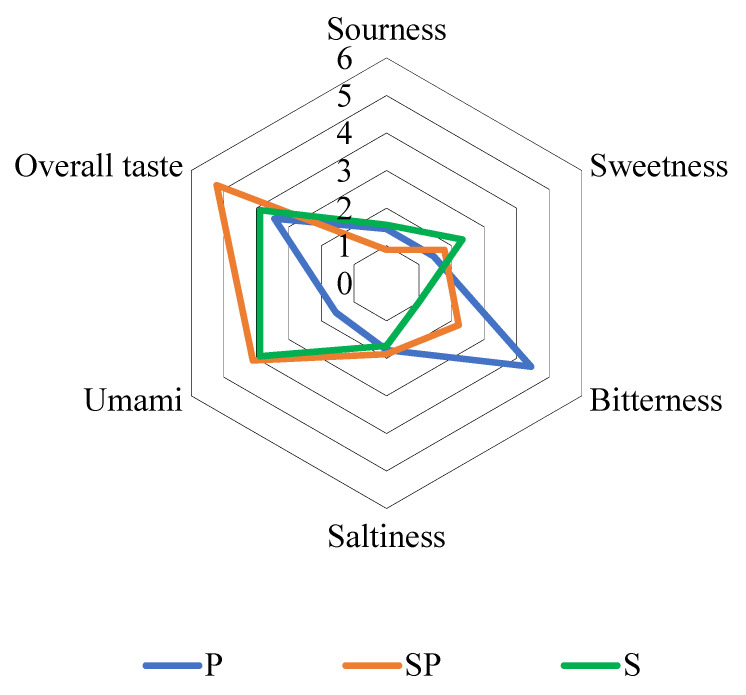
Results of the sensory evaluation.

The results of the sensory evaluation are shown in Figure 1. After adding prickly ash, the overall taste of the stewed sheep tail fat significantly improved (from 3.89 to 5.22). This may be because the addition of prickly ash improved the bitter taste of the SP sample, making the overall taste improve. Yang et al. analyzed the compounds in pepper through LC-MS; 15 key compounds were selected by a combination with with omics analysis technology and most of them had a low bitter recognition threshold by the sensory evaluation [20]. The sensory evaluation results of the P sample showed that the bitter taste of prickly ash was stronger. Therefore, we found that improving the overall taste of stewed sheep tail fat may be related to the bitter taste of prickly ash. Nodake et al. used an electronic tongue to analyze the taste changes of meat in the curing process and found that the bitterness and saltiness were significantly related to the curing time (*R* = 0.98). The bitterness value was also significantly related to the content of bitter amino acids (*R* = 0.96) [21]. Therefore, they inferred that the increase in the bitterness or saltiness compounds was the reason for the characteristic flavor of cured meat.

### 3.2. Compound Identification Results

**Figure 2:**
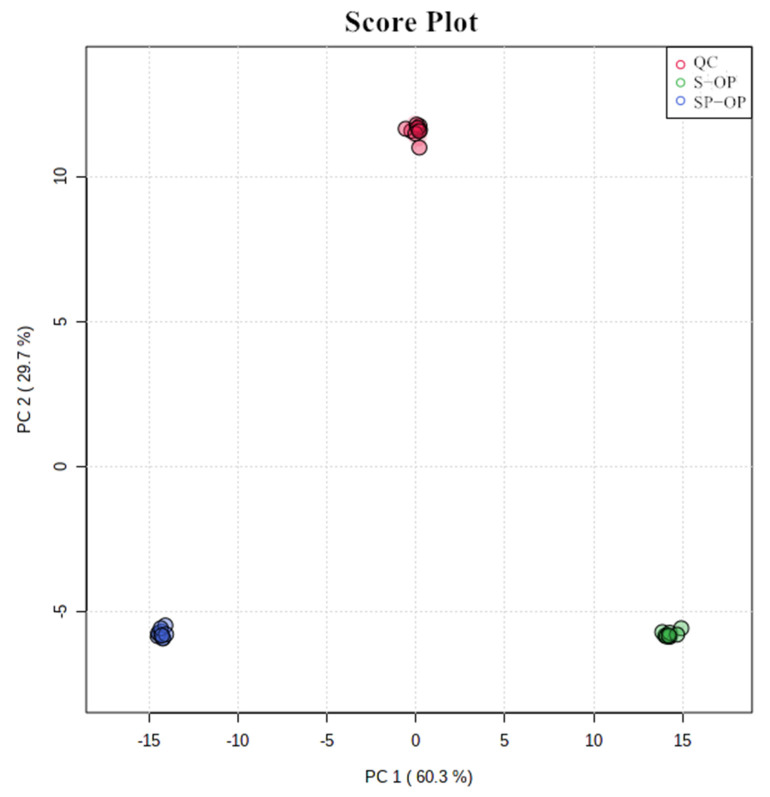
PCA score plots of the soup samples and the QC sample.

According to the matching results of the database, 99 compounds (both ESI^+^ and ESI^−^) were identified including amino acids (14), nucleotides (17), organic acids (16), sugars (3), peptides (2), fatty acids (6), flavonoids (10) and others (31) (Table S3). In order to check the data stability, missing values were deleted and then the data were normalized to reduce any systematic bias or technical variation. Compounds having more than 50% missing values were not used in the subsequent analysis. The PCA results between the QC sample and the soup samples (SP-OP, S-OP) showed a good separation (Figure 2), which showed the reliability of the data [22]. Indirectly, the LC and MS conditions were suitable for the measurement of the samples confirmed in this work.

### 3.3. Marker Compound Analysis

**Figure 3:**
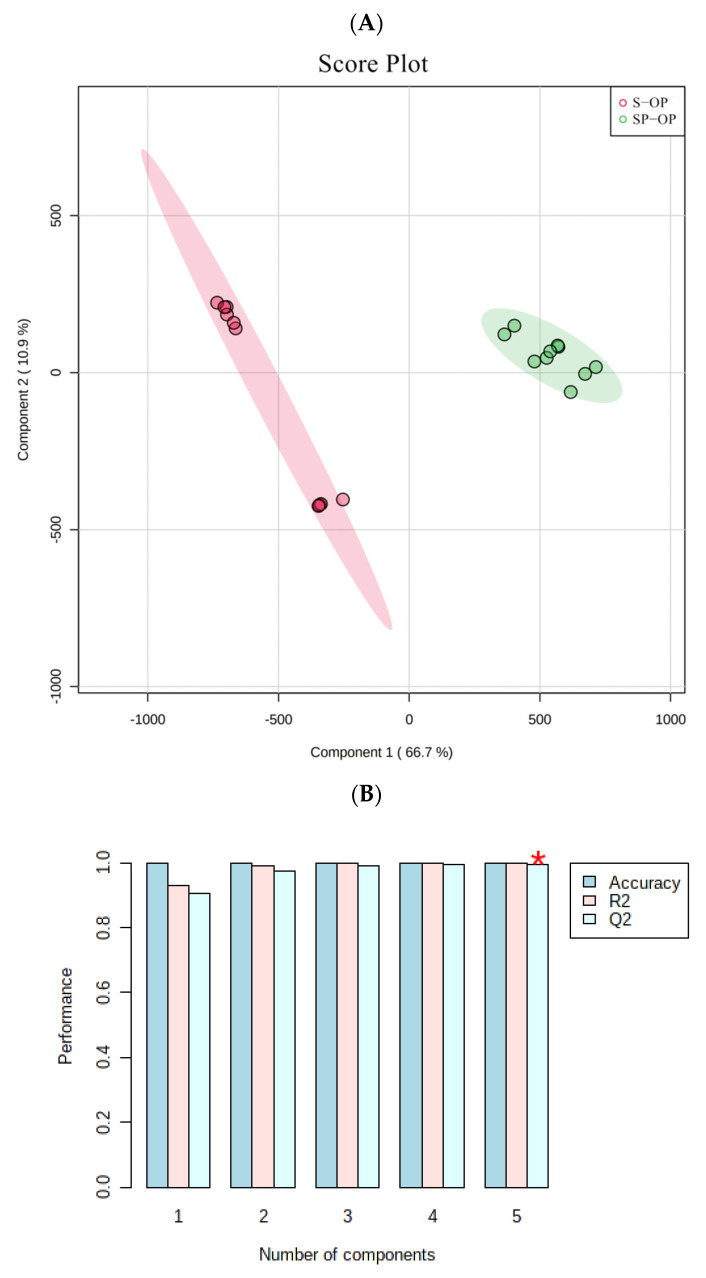

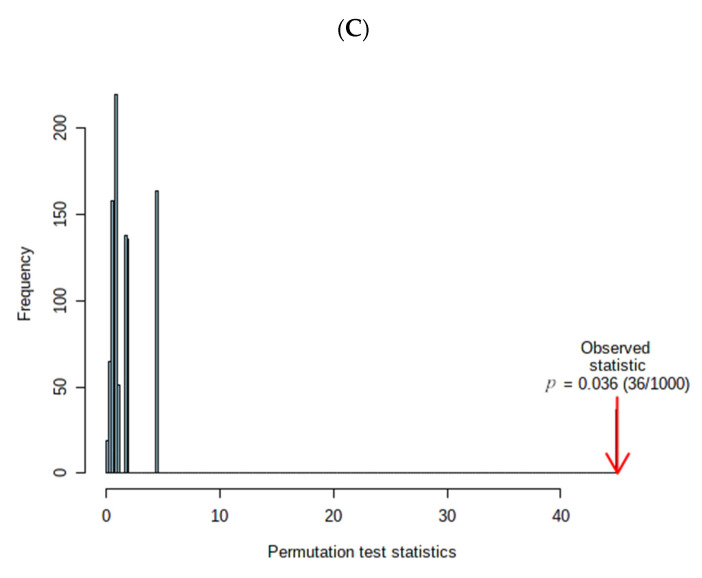
(**A**) PLS-DA score plots of the soup samples (SP-OP and S-OP). (**B**) Cross validation results (The best classifier was indicated by the red asterisk). (**C**) Permutation test results (1000 cycles (*p* < 0.05)).

According to the semi-quantitative results, a PLS-DA model was established to select the marker compounds taking the soup samples as an example (Figure 3). In this model, distinct separated clusters were observed between the compounds of the two sample groups (Figure 3A). The parameters of the PLS-DA model (*R*^2^ = 0.997, *Q*^2^ = 0.984) obtained can be seen in Figure 3B where *R*^2^ and *Q*^2^ were ≥ 0.5, suggesting that the model was stable and reliable [23]. The permutation test of the PLS-DA model is shown in Figure 3C; overfitting did not occur in the PLS-DA model. Based on the PLS-DA model, the marker compounds from the soup, fat block and upper oil layer samples were selected by VIP > 1, FC > 2 and *p* < 0.05, as summarized in Table 1.

Three marker compounds were selected in the soup samples; 5′-GMP, malic acid and tanshinone IIA (Table 1). 5′-GMP is an important umami enhancer substance and, when used together with 5′-IMP, it has a strong effect on the umami characteristics. It can also reduce the addition of NaCl in food [24]. Malic acid was identified as a marker compound. This may be because the addition of prickly ash promoted the release of malic acid in sheep tail fat or because malic acid exists in prickly ash, which increased in the SP-OP sample by superposition. Huang et al. identified malic acid in the taste components of prickly ash extracted by ultrasonic-assisted ethanol, which confirmed that malic acid existed in prickly ash [25]. Tanshinone IIA was detected in sheep tail fat, which might result in the intake of *Salvia miltiorrhiza Bunge* during the predation process and the production of tanshinone IIA through the catabolism in vivo that has not been completely metabolized. However, up to the present time, there is no literature on its taste characteristics, only studies on its anti-tumor effect [26]. Therefore, there is no detailed discussion here.

Three marker compounds were selected from the fat block samples after stewing; guanosine, 5′-AMP and inosine (Table 1). Nucleosides exist widely in food; inosine, guanosine and adenosine were determined to be major nucleosides accounting for 60% of the total nucleosides in the Ganoderma species [27]. Nucleosides are usually not accepted by consumers because they have a bad flavor substance. However, with the deepening of flavor research, it has been found that the taste characteristics of food are not affected by a single taste and that there are certain interactions among tastes. Therefore, several unfriendly tastes such as bitterness and sourness may also play an important role in the overall taste of food products. Ismail et al. used an electronic tongue to study the effects of a temperature–time combination on the non-volatile components and taste characteristics of beef and found that 5′-AMP and 5′-GMP played an important role in the presentation of flavor [28]. Barido et al. studied the effect of different concentrations of Cordyceps militaris mushrooms on the taste of commercial broilers. The results showed that 2% could enrich the taste-related compounds, particularly an increase in 5′-AMP and umami-related free amino acid compounds [29].

A total of five compounds were selected in the upper oil layers samples; stearic acid, fumaric acid, 18β-glycyrrhetinic acid, methyl jasmonatepure and 2-*N*-heptylfuran (Table 1). Stearic acid widely exists in animal fat and fat oxidation can improve food flavor. Methyl jasmonatepure and 2-*N*-heptylfuran were analyzed mostly as volatile compounds and we concluded that methyl jasmonatepure was mainly floral and fruity whereas 2-*N*-heptylfuran was mainly roasted nut and fatty (http://www.thegoodscentscompany.com/search2.html (accessed on 25 September 2021). This work mainly focused on the taste compounds; that is, non-volatile water-soluble compounds. The two compounds were, therefore, not discussed in detail although they were detected through the LC-MS system. 18β-glycyrrhetinic acid mainly comes from licorice but it has been detected in sheep tail fat. This may be because sheep can feed on licorice and the glycyrrhizic acid in licorice degrades into 18β-glycyrrhetinic acid in the sheep body. He et al. found certain residues in different tissues of rats fed with nutritious soup, which further verified the factual basis for the detection of 18β-glycyrrhetinic acid in sheep tail fat [30]. As a sweet substance, 18β-glycyrrhetinic acid was found with a content of 0.529 mg/100 mg and it was 166 times sweeter than sucrose, which was researched from derris reticulata (a Thai plant) [31].

The above analysis indicated the addition of prickly ash promoted the release of a few compounds. This might have been due to the presence of these compounds in prickly ash or the interaction during the stewing process that promoted the release of these compounds. Yu et al. studied the effect of natural spices on the flavor substances of stewed Wuding chicken. The results showed that the content of the total fatty acids, water-soluble small molecular weight compounds and volatile flavor substances in the treatment group was significantly higher than that in the control group (*p* < 0.05) [32]. It could, therefore, be concluded that natural spices had a significant effect on the flavor precursor substances of boiled Wuding chicken. This also confirmed the significance of adding prickly ash in stewing sheep tail fat in this work.

### 3.4. Taste Characteristics Analysis Results

Through the analysis of the marker compounds and combined with the results reported in the previous literature, 7 compounds (nucleotides and organic acids) were selected, which were 5′-GMP and malic acid in the OP samples; inosine, 5′-AMP and guanosine in the OB samples; and fumaric acid and 18β-glycyrrhetinic acid in the OU samples.

**Figure 4:**
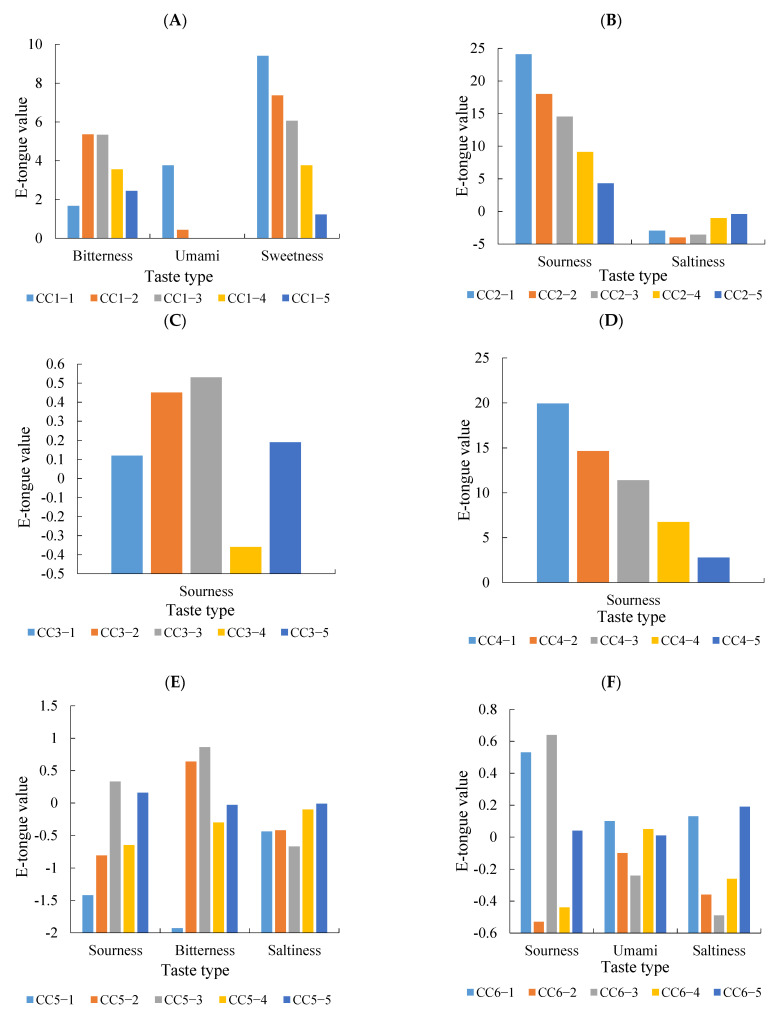

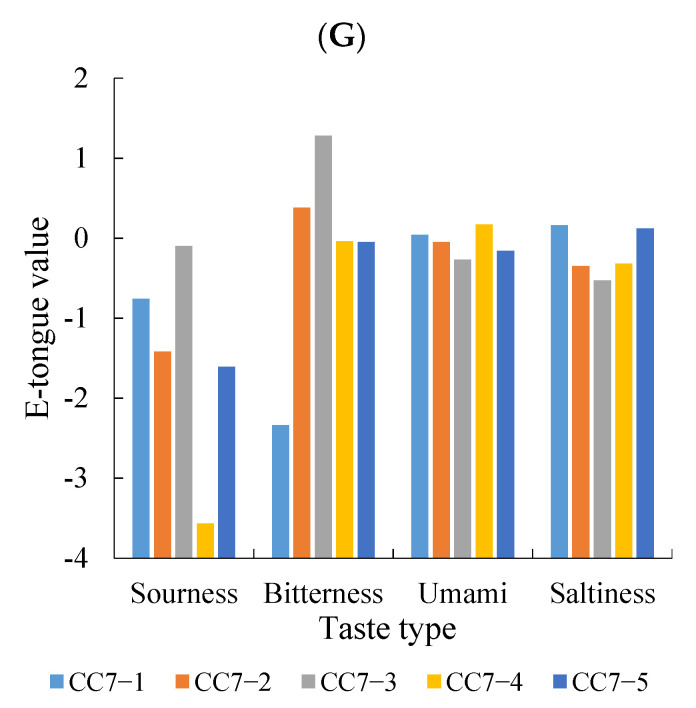
Electronic tongue results of the standard compounds ((**A**) electronic tongue results of 5′-GMP in different concentration (CC1-1: 378.25 μg/mL, CC1-2: 151.30 μg/mL, CC1-3: 75.65 μg/mL, CC1-4: 37.83 μg/mL, CC1-5: 15.13 μg/mL); (**B**) electronic tongue results of malic acid in different concentration (CC2-1: 58.75 μg/mL, CC2-2: 23.50 μg/mL, CC2-3: 11.75 μg/mL, CC2-4: 5.88 μg/mL, CC2-5: 2.35 μg/mL); (**C**) electronic tongue results of inosine in different concentration (CC3-1: 2889.97 μg/mL, CC3-2: 1155.99 μg/mL, CC3-3: 577.99 μg/mL, CC3-4: 289.00 μg/mL, CC3-5: 115.60 μg/mL); (**D**) electronic tongue results of 5′-AMP in different concentration (CC4-1: 160.08 μg/mL, CC4-2: 64.03 μg/mL, CC4-3: 32.02 μg/mL, CC4-4: 16.01 μg/mL, CC4-5: 6.40 μg/mL); (**E**) electronic tongue results of guanosine in different concentration (CC5-1: 250.27 μg/mL, CC5-2: 100.11 μg/mL, CC5-3: 50.05 μg/mL, CC5-4: 25.03 μg/mL, CC5-5: 10.01 μg/mL); (**F**) electronic tongue results of fumaric acid in different concentration (CC6-1: 0.40 μg/mL, CC6-2: 0.16 μg/mL, CC6-3: 0.08 μg/mL, CC6-4: 0.04 μg/mL, CC6-5: 0.02 μg/mL); (**G**) electronic tongue results of 18β-glycyrrhetinic acid in different concentration (CC7-1: 32.85 μg/mL, CC7-2: 13.14 μg/mL, CC7-3: 6.57 μg/mL, CC7-4: 3.29 μg/mL, CC7-5: 1.31 μg/mL).

**Figure 5:**
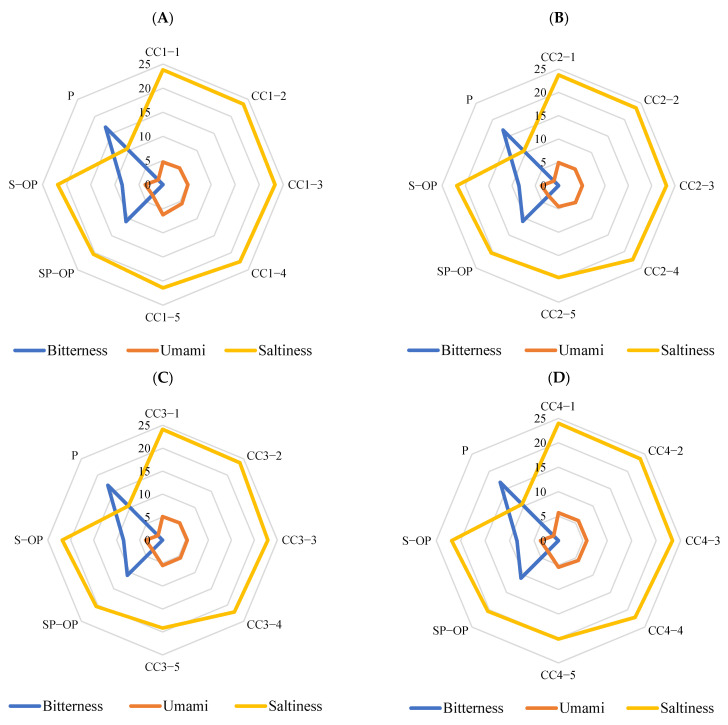

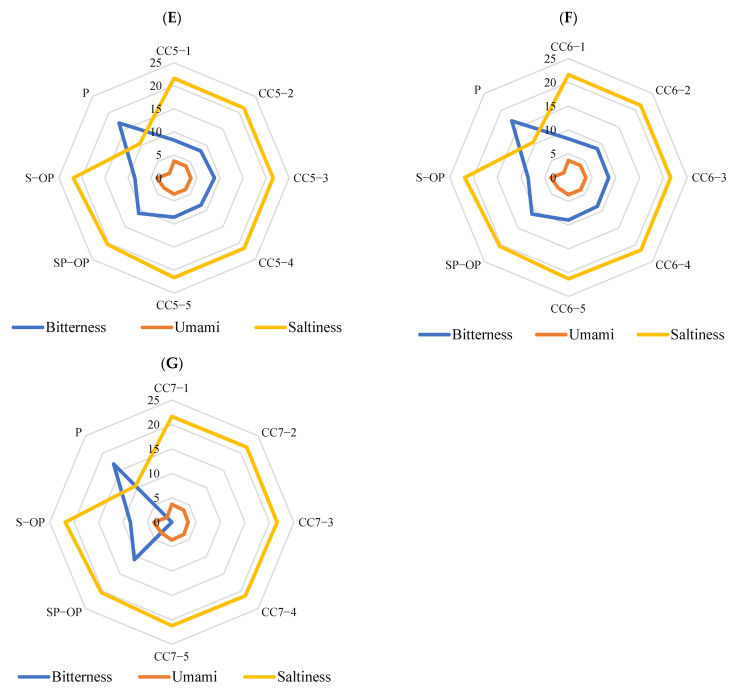
Analysis results of the additional experiment ((**A**) addition experiment results of 5′-GMP; (**B**) addition experiment results of malic acid; (**C**) addition experiment results of inosine; (**D**) addition experiment results of 5′-AMP; (**E**) addition experiment results of guanosine; (**F**) addition experiment results of fumaric acid; (**G**) addition experiment results of 18β-glycyrrhetinic acid).

The electronic tongue analysis results of the seven compounds are shown in Figure 4. The 5′-GMP (Figure 4A) was mainly umami, sweetness and bitterness at a higher concentration multiple (CC1-1 and CC1-2); with the decrease of concentration, the umami taste disappeared, bitterness and sweetness decreased and there was a certain correlation between the decrease of sweetness and the change of concentration. We observed that a few compounds did not have single taste properties and the taste properties were related to the concentration of the compounds and the taste interaction. When umami and sweetness values are higher, bitterness may be inhibited [33]; if the umami and sweetness values decrease at low concentrations, a bitter taste can be perceived. This could explain why there was no correlation between the bitterness value of 5′-GMP and the change of concentration. However, with a decrease of the concentration, the response of the bitterness value will also decrease gradually. This might be related to the threshold of the taste compound [34]. Malic acid (Figure 4B) and 5′-AMP (Figure 4D) have a sour taste, which can improve the overall taste by adjusting the sourness taste of food. Choi and Garza explored the application of miracle fruit (with a sour taste) in different foods products [35]. A Tukey post-hoc test showed that the pre-to-post increments for the overall flavor and texture likings in yogurt and the overall and flavor likings in apple using miracle fruit products were significantly higher than using other products (*p* < 0.05). 18β-glycyrrhetinic acid has been reported as having a strong sweetness taste [31] but it was not detected on the electronic tongue in this experiment (Figure 4G). This may be related to the concentration of the experimental sample and the response of the sweetness sensor.

### 3.5. Additional Experiment

An additional test was used to verify the taste effect of the compounds in stewed sheep tail fat. Seven compounds were added to the S-OP sample individually to explore the taste contribution. The analysis results of the electronic tongue are shown in Figure 5. The addition of 5′-GMP (Figure 5A) improved the taste value of the stewed sheep tail fat, which was consistent with the results reported that nucleotides function as taste enhancers [36]. Zhan et al. analyzed the non-volatile flavor compounds through a sensory evaluation and a PLS-DA. 5′-GMP and a few amino acids had a significant positive effect on taste so it was speculated that they were the main components affecting the umami taste of chicken soup. The addition of 5′-GMP also increased the saltiness and significantly reduced the bitterness taste [37]. The addition of malic acid (Figure 5B) improved the umami and saltiness taste in stewed sheep tail fat, which might be used as an acid additive to regulate the taste characteristics. Studies have shown that the umami taste is related to the pH value so the addition of malic acid may also improve the taste by adjusting the pH value [38]. The additional effect of 5′-AMP (Figure 5D) was the same as that of malic acid and the standard compound was the sour taste. 5′-AMP could enhance the umami taste of the sample except for its sour taste. Studies have shown that 5′-AMP can be transformed into 5′-IMP to improve the umami taste [39]. Inosine (Figure 5C) had a slight sourness response in the electronic tongue but when added to the sample, it also improved the umami and saltiness taste. Guanosine (Figure 5E), fumaric acid (Figure 5F) and 18β-glycyrrhetinic acid (Figure 5G) had no significant effect on the sheep tail fat samples. This might be related to the weak taste intensity of the compound itself. Although 18β-glycyrrhetinic acid has been reported to have a sweet taste [31], it was not detected in this experiment, which may be related to the concentration of the compound or the response of the sensor.

## 4. Conclusions

A total of 99 compounds were detected in stewed sheep tail fat samples by LC-QTOF-MS/MS. The semi-quantitative results showed that the addition of prickly ash resulted in a difference of the compounds in sheep tail fat. A PLS-DA was performed to predict 11 marker compounds associated with the taste of stewed sheep tail fat. An additional experiment was conducted to explore the taste contribution of compounds (three organic acids and four nucleotides) to stewed sheep tail fat. The electronic tongue results showed that 5′-GMP, malic acid, inosine and 5′-AMP had a taste effect on stewed sheep tail fat, which improved the umami and saltiness taste and reduced the bitterness taste. This work confirmed the contribution of prickly ash to the taste effect of stewed sheep tail fat and provides theoretical guidance for the processing of sheep tail fat.

## Figures and Tables

**Table 1:** Information of the marker compounds.

Type	Compound	MW	RT (min)	Ion	VIP	FC	*p*-Value
Soups	Tanshinone IIA	294.18918	55.77	[M + H]^+^	1.06	2.59	<0.01
	Malic acid	134.02227	1.44	[M − H]^−^	1.26	3.86	<0.01
	5′-GMP	363.05844	1.94	[M − H]^−^	1.94	4.07	<0.01
Fat blocks	Guanosine	283.09202	2.86	[M − H]^−^	1.65	16.29	<0.01
	5′-AMP	347.06336	3.14	[M − H]^−^	1.76	34.87	<0.01
	Inosine	268.08227	1.75	[M + H]^+^	1.85	55.05	<0.01
Upper oils	18β-glycyrrhetinic acid	470.29115	54.97	[M − H]^−^	1.04	2.52	<0.01
	Fumaric acid	115.99666	1.36	[M − H]^−^	1.03	2.68	<0.01
	Methyl jasmonatepure	224.03637	50.16	[M − H]^−^	1.24	3.50	<0.01
	2-*N*-heptylfuran	166.04874	1.43	[M − H]^−^	1.38	4.54	<0.01
	Stearic acid	284.14545	51.37	[M − H]^−^	1.54	15.84	<0.01

## Supplementary Materials

The following are available online at https://www.mdpi.com/article/10.3390/foods10112709/s1, Table S1: Classification of quantitative compounds and internal standard compounds, Table S2: Addition experiment with compounds in different concentration, Table S3: Different types of identified compounds.
