# Supplementary material for: The Effect of Prickly Ash (Zanthoxylum bungeanum Maxim) on the Taste Perception of Stewed Sheep Tail Fat by LC-QTOF-MS/MS and a Chemometrics Analysis
Source: Foods. 2021 Nov 5;10(11):2709. doi: 10.3390/foods10112709 (PMC8622103; doi:10.3390/foods10112709)
Supplement: Supplementary file 1 [file foods-10-02709-s001.zip › foods-1435742-supplementary.pdf]

Effect of prickly ash (*Zanthoxylum bungeanum* Maxim) on the taste perception of stewed sheep tail fat by LC-QTOF-MS/MS and chemometrics analysis

Yan Huang, Dandan Pu, Zhilin Hao, Xiao Yang and Yuyu Zhang*

[1] Beijing Key Laboratory of Flavor Chemistry, Beijing Technology and Business University, Beijing 100048, China
* Correspondence: yuyu zhang; zhangyuyu@btbu.edu.cn; Tel.: 15001349082. e-mail@e-mail.com; E-mail addresses of co-authors: huangyan_916@163.com (Yan Huang), 18518351472@163.com (Dandan Pu), hzl15716324037@163.com (Zhilin Hao), yangxiao@btbu.edu.cn (xiao yang)

Table S1. Classification of quantitative compounds and internal standard compounds.

Internal standard compounds	Quantitative compounds
Flavone	1-methyladenosine, inosine, ADP, 5'-CMP，guanosine, 5'-GMP，guanosine-3', 5'-cyclic monophosphate, uridine, UMP, adenosine, 5'-AMP, cyclic AMP, (-)-Catechin hydrate, quercetin, rutin, isoquercitrin, isorhamnetin 3-O-neohesperosine, genistein, emodin, daidzein, kaempferol-3-O-rutinoside, hyperoside, cynaroside, astragalin
L-Theanine	Glu, Cys, Arg, Leu, Asn, Asp, Val, Leu, succinic acid, fumaric acid, 2-Methylpropanedioic acid, citric acid, taurine, malic acid, Glu-Val，glutathione, acrylamide, betaine, L-carnitine, D(+)-Glucopyranose 6-phosphate, D(+)-Glucose, D-pantothenic acid , N-(Phosphonomethyl)glycine 2-propylamine
1,2-Dichlorobenzene	2,6-dihydroxypurine, cytosine, 6-hydroxypurine, guanine, adenine, pyroglutamic acid, 2-Pyrrolidinone, protocatechualdehyde, 3,4-Dihydroxybenzoic acid, benzothiazole, methyl eugenol, trigonelline, bis(2-ethylhexyl) phthalate, dibutyl phthalate, diallyl phthalate, diethyl phthalate, 3-Hydroxy-2-methyl-4H-pyran-4-one, nicotinic acid
Methyl palmitate	linolenic acid, linoleic acid, stearic acid, palmitic acid, arachidonic acid, N-(phenylMethyl)-, nerolidol
Silibinin	D-sucrose, cholic acid, glycocholic acid, ganoderic acid Y, chlorogenic acid, cryptochlorogenic acid, 18β-glycyrrhetinic acid, β-estradiol, tanshinone IIA, triphenyl phosphate, promethazine, LSD, quinestrol
gamma-Nonanolactone	Phe, Tyr, Pro, Trp, His, ferulic acid, caffeic acid, desmedipham, 2-N-heptylfuran, isojasmone, alpha-bisabolol, methyl jasmonatepure, capsaicin, nicotinamide

Table S2. Addition experiment with compounds in different concentration.

Name	Concentration (μg/mL)				
Multiple	5	2	1	0.5	0.2
5'-GMP	CC1-1	CC1-2	CC1-3	CC1-4	CC1-5
	378.25	151.30	75.65	37.83	15.13
Malic acid	CC2-1	CC2-2	CC2-3	CC2-4	CC2-5
	58.75	23.50	11.75	5.88	2.35
Inosine	CC3-1	CC3-2	CC3-3	CC3-4	CC3-5
	2889.97	1155.99	577.99	289.00	115.60
5'-AMP	CC4-1	CC4-2	CC4-3	CC4-4	CC4-5
	160.08	64.03	32.02	16.01	6.40
Guanosine	CC5-1	CC5-2	CC5-3	CC5-4	CC5-5
	250.27	100.11	50.05	25.03	10.01
Fumaric acid	CC6-1	CC6-2	CC6-3	CC6-4	CC6-5
	0.40	0.16	0.08	0.04	0.02
18β-Glycyrrhetinic acid	CC7-1	CC7-2	CC7-3	CC7-4	CC7-5
	32.85	13.14	6.57	3.29	1.31

Table S3. Different types of identified compounds.

Types	Compounds
Amino acids (14)	Phe, Glu, Cys, pyroglutamic acid, Arg, Tyr, Leu、Pro, Trp, Asn, Asp, Val, Leu, His
Nucleotides (17)	1-methyladenosine, 2,6-dihydroxypurine, 5'-CMP、cytosine, inosine, 6-hydroxypurine, ADP, guanosine, 5'-GMP、guanine, guanosine-3', 5'-cyclic monophosphate, uridine, UMP, adenosine, cyclic AMP, 5'-AMP、adenine
Organic acids (16)	18β-glycyrrhetinic acid, D-pantothenic acid, ferulic acid, cholic acid, fumaric acid, glycocholic acid, succinic acid, 2-methylpropanedioic acid, caffeic acid, ganoderic acid Y, chlorogenic acid, citric acid, taurine, malic acid, nicotinic acid, cryptochlorogenic acid
Sugars (3)	D(+)-Glucopyranose 6-phosphate, D(+)-Glucose, D-sucrose
Peptides (2)	Glu-Val, glutathione
Fatty acids (6)	N-(phenylMethyl)-, arachidonic acid, linolenic acid, linoleic acid, palmitic acid, stearic acid
Flavonoids (11)	(-)-Catechin hydrate, quercetin, genistein, hyperoside, kaempferol-3-O-rutinoside, rutin, cynaroside, isoquercitrin, isorhamnetin 3-O-neohesperosine, astragalin
Others (30)	desmedipham, 2-Pyrrolidinone, 2-N-heptylfuran, protocatechualdehyde, 3,4-Dihydroxybenzoic acid, N-(Phosphonomethyl)glycine 2-propylamine, benzothiazole, acrylamide, nerolidol, β-estradiol, emodin, tanshinone IIA, methyl eugenol, isojasmone, alpha-bisabolol, trigonelline, methyl jasmonatepure, capsaicin, bis(2-ethylhexyl) phthalate, dibutyl phthalate, diallyl phthalate, diethyl phthalate, triphenyl phosphate, LSD, 3-Hydroxy-2-methyl-4H-pyran-4-one, quinestrol, betaine, nicotinamide, promethazine, L-carnitine
